# Teachers Can Make a Difference in Bullying: Effects of Teacher Interventions on Students’ Adoption of Bully, Victim, Bully-Victim or Defender Roles across Time

**DOI:** 10.1007/s10964-022-01674-6

**Published:** 2022-09-02

**Authors:** Christoph Burger, Dagmar Strohmeier, Lenka Kollerová

**Affiliations:** 1 Faculty of Psychology, Department of Developmental and Educational Psychology, University of Vienna, Vienna, Austria; 2 Faculty of Psychology, Department of Cognition, Emotion, and Methods in Psychology, University of Vienna, Vienna, Austria; 3 Department of Psychology and Psychodynamics, Division of Psychological Methodology, Karl Landsteiner University of Health Sciences, Krems, Austria; 4 School of Medical Engineering and Applied Social Sciences, University of Applied Sciences Upper Austria, Linz, Austria; 5 Center for Learning Environment and Behavioral Research, University of Stavanger, Stavanger, Norway; 6 Czech Academy of Sciences, Institute of Psychology, Prague, Czech Republic

**Keywords:** Bullying, Teacher interventions, Teacher strategies, Anti-bullying, Disciplinary sanctions, Group discussions

## Abstract

School bullying is a serious problem worldwide, but little is known about how teacher interventions influence the adoption of bullying-related student roles. This study surveyed 750 early adolescents (50.5% female; average age: 12.9 years, *SD* = 0.4) from 39 classrooms in two waves, six months apart. Peer ratings of classmates were used to categorize students to five different bullying-related roles (criterion: >1 *SD*): bully, victim, bully-victim, defender, and non-participant. Student ratings of teachers were used to obtain class-level measures of teacher interventions: non-intervention, disciplinary sanctions, group discussion, and mediation/victim support. Controlling for student- and class-level background variables, two multilevel multinomial logistic regression analyses were computed to predict students’ bullying-related roles at wave 2. In the static model, predictors were teacher interventions at wave 1, and in the dynamic model, predictors were teacher intervention changes across time. The static model showed that disciplinary sanctions reduced the likelihood of being a bully or victim, and group discussion raised the likelihood of being a defender. Mediation/victim support raised the likelihood of being a bully. The dynamic model complemented these results by indicating that increases in group discussion across time raised the likelihood of being a defender, whereas increases in non-intervention across time raised the likelihood of being a victim and reduced the likelihood of being a defender. These results show that teacher interventions have distinct effects on students’ adoption of bullying-related roles and could help to better target intervention strategies. The findings carry practical implications for the professional training of prospective and current teachers.

## Introduction

Healthy and safe schools play a critical role in positive youth development (D’Urso et al., 2021). However, bullying affects students worldwide and has been shown to have potentially serious and long-lasting effects on health, well-being, academic performance, and occupational success (Burger & Bachmann, 2021), not only for victims but for all students involved (Hysing et al., 2021). This is particularly true for students in early adolescence where bullying peaks (Swearer et al., 2017) and where students are even at a higher risk for developing mental health disorders due to changes in social dynamics, biology, and cognition (Salmivalli et al., 2021). It has been demonstrated that teachers can stop bullying (Troop-Gordon & Ladd, 2015) and that teacher interventions have a central role in the fight against bullying (Colpin et al., 2021). But there is debate about which teacher interventions are effective (Bauman et al., 2021) because previous studies have largely relied on cross-sectional and variable-centered approaches. It is unclear what kind of teacher interventions are followed by actual changes in bullying-related student behaviors and whether the effects of teacher interventions differ for bullies, victims, bully-victims, defenders and bystanders. In addition, previous research has typically used teacher self-reports to assess teacher interventions and student self-reports to measure bullying-related behaviors, both of which are susceptible to social desirability bias (Wachs et al., 2019). The present study aims to overcome these methodological limits by applying a person-centered approach using multiple informants (both teacher and student behavior were based on multiple observations of students in each class). Using a longitudinal research design, the present study aims to elucidate whether and to what extent different teacher interventions have differential effects on the adoption of bullying-related roles among adolescents over a period of six months.

### Bullying-Related Roles

Bullying has been identified as a complex group process that unfolds from social interactions in the classroom where individuals adopt distinct bullying-related roles (Pouwels et al., 2018), including bullies, victims, but also bully-victims, non-participants, and defenders. Students who bully others are more often male than female (Smith et al., 2019), they are more powerful than their targets (Menesini & Salmivalli, 2017) and tend to use aggression to maintain and enhance their social status (Košir, et al., 2021). Victims tend to have no or very few friends (Stefanek et al., 2017) and usually withdraw without fighting back, making them attractive targets for long-time victimization (Brendgen & Poulin, 2018). A growing body of research has identified a group of students who are both bullies and victims. These bully-victims have been classified as a particularly high-risk group because they suffer from the negative effects associated with both roles (Sung et al., 2018) and exhibit even higher levels of psychological maladjustment than bullies or victims (Yang et al., 2016). The vast majority of students are neither bullies nor victims, but bystanders (Callaghan et al., 2019). These students are usually present as witnesses in bullying episodes, but instead of helping the victims they passively watch the bullying unfold or empower the bullies through explicit social rewards like laughing (Troop-Gordon et al., 2019). Some students (most likely girls) defend victims by attempting to stop the bullies or comfort the victimized students (Yun, 2019). Even when defenders are not able to stop the bullying, victims who are defended by their peers show lower levels of anxiety and depression (Salmivalli, 2014) and higher feelings of school belonging (Laninga-Wijnen et al., 2022).

### Teacher Interventions

Teachers function not only as educators, but also as socialization agents and classroom managers (De Luca et al., 2019), and they have a legal obligation to protect their students from harm. While teachers report being highly likely to intervene when bullying happens, student surveys indicate the opposite (Bradshaw et al., 2007). A lack of teacher interventions is problematic on many levels. Bullies might interpret it as a tacit approval of the bullying (Saarento et al., 2015) and learn that bullying does not result in disciplinary consequences. Bystanders might refrain from reporting bullying or from defending victims in the future, and victims might receive the message that they are not safe at school (Mucherah et al., 2018).

Teachers’ active responses to bullying typically include a variety of interventions (Kollerová et al., 2021). Teachers typically prefer authority-based disciplinary interventions against bullying (Burger et al., 2015). Disciplinary interventions of moderate severity that are delivered in a non-hostile, warm atmosphere (i.e., authoritative intervention style; Gee et al. 2021) have been associated with lower school bullying (Cornell & Huang, 2016). Teachers can employ such authority-based interventions to send a clear message of disapproval (Saarento et al., 2013), which might reduce bullying by increasing moral engagement in peers (Campaert et al., 2017). Disciplinary sanctions are not only an integral part of many anti-bullying programs, such as the KiVa anti-bullying program (Garandeau et al., 2021), the Olweus bullying prevention program (Limber et al., 2018) and the ViSC social competence program (Strohmeier et al., 2021), but their effectiveness is corroborated by a recent meta-analysis on the effectiveness of intervention components in anti-bullying programs (Gaffney et al., 2021).

Considering that bullying is a social process (Farmer et al., 2019), teacher-facilitated discussions might be an effective way for the whole class to work on bullying prevention collaboratively. Such discussions could raise awareness on the serious consequences of bullying and the crucial role of bystanders. They might lead to higher moral responsibility in the class and lower social acceptability of bullying behavior and, in turn, increase the likelihood of being a defender (Jungert et al., 2016). A recent meta-analysis showed that discussions about bullying were associated with lower bullying rates when insights and opinions were gained naturally by the students but not when they were unilaterally imposed on the students by the teachers (Gaffney et al., 2021).

Another teacher response sometimes advocated for resolving bullying cases is mediation, which is understood as a non-punitive conflict resolution technique where both parties are given the opportunity to express their opinions (Morese et al., 2018). Typically, two students voluntarily attempt to resolve their conflicts with the help of a teacher as a neutral third party. Applying mediation to bullying cases is problematic because it is based on the erroneous assumption that victims and bullies can have the same weight in representing their interests (Rigby, 2012). Because bullies might have good social knowledge and theory of mind skills, they might be able to talk their way out (Rawlings, 2019). In line with these concerns, studies that addressed effectiveness of mediation on changing bullying-related behaviors found no effects (Campaert et al., 2017).

Teachers may decide to intervene by supporting victims (Berkowitz & Benbenishty, 2012), such as by providing emotional support, increasing the victims’ assertiveness in dealing with aggressive peers, or connecting them with pro-social students (Rigby, 2012). Although a recent meta-analysis revealed that victim support reduced bullying (Gaffney et al., 2021), teachers tend to focus on bullies and neglect victims (Burger et al., 2015).

While the existing research provided key insights into the role of teachers in bullying interventions, it largely relied on cross-sectional and variable-centered approaches (Bayram Özdemir et al., 2021; Campaert et al., 2017; van Aalst et al., 2021), so it is not clear whether teacher interventions were followed by changes in student bullying behaviors and whether the effects differed for students with distinct bullying-related roles, including the most vulnerable group of bully-victims (Yang et al., 2016). The research has typically used teacher self-report to assess teacher interventions and student self-reports to measure bullying-related student behaviors, which are both susceptible to social desirability bias (Wachs et al., 2019). To date, no study has used a dynamic approach to address the impact of teacher behavior change across the school year (Morgan et al., 2014) on bullying-related student outcomes, although the conventional static approach and the dynamic approach have been found to provide complementary information (Nguyen et al., 2020). To markedly advance the current knowledge and better inform teacher education, a longitudinal person-centered approach with highly valid measures (e.g., peer ratings for bullying-related behavior, student reports for teacher interventions) and analytical methods (longitudinal; Troop-Gordon et al., 2019; person-oriented; Burger & Bachmann, 2021) is warranted.

## Current Study

**Fig. 1:**
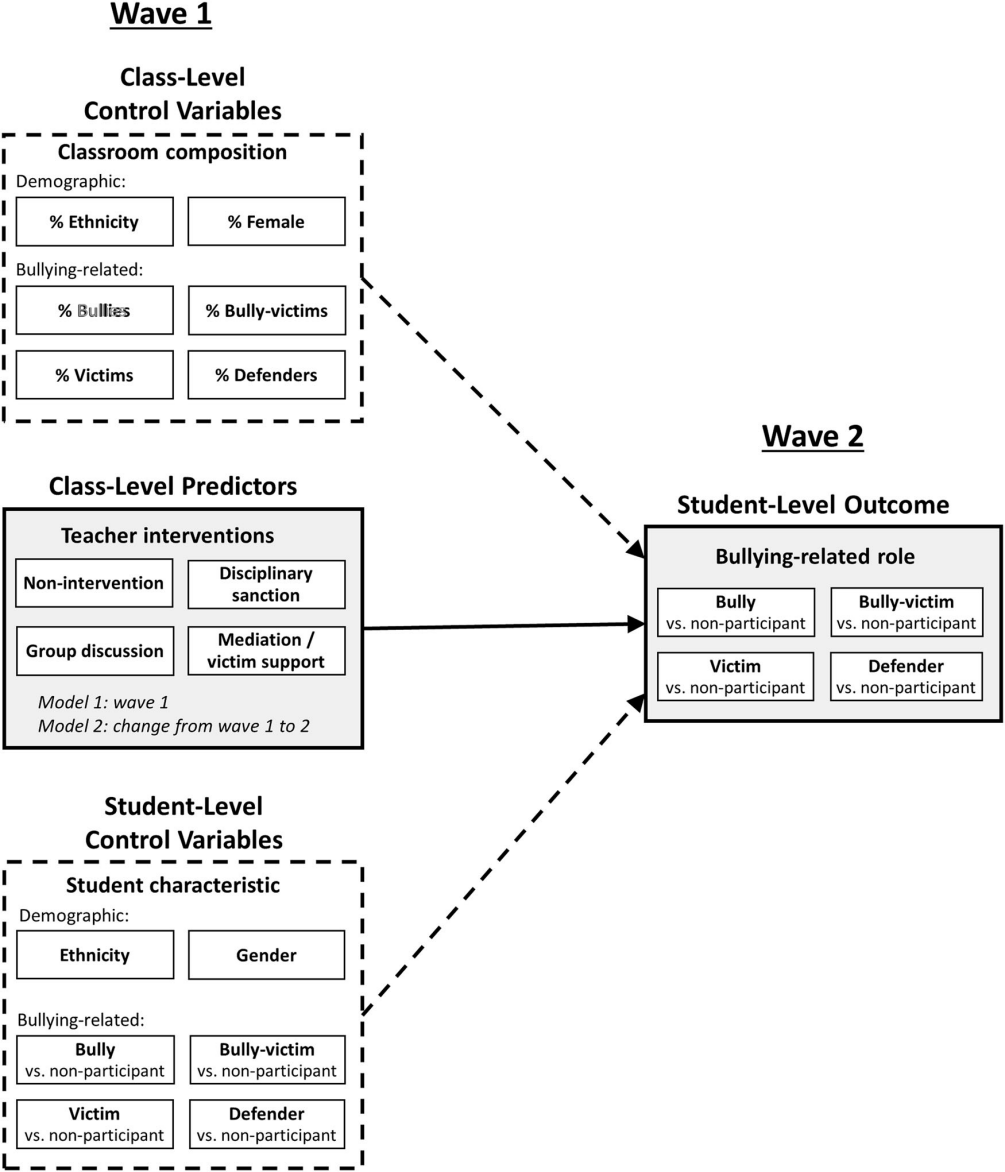
Multi-level framework of teacher interventions influencing bullying-related role adoption while controlling for individual background and class composition

Although teachers play an important role in bullying prevention, it is unclear which teacher interventions are effective in changing bullying-related roles in early adolescent students across time. A longitudinal person-oriented framework (see Fig. 1) with two time points six months apart (the last wave being at the end of the school year) was utilized, adjusting for relevant control variables (demographic and bullying-related student characteristics at wave 1) on the student- and classroom-level. In addition to a static longitudinal analytical approach, which relates teacher interventions at wave 1 to future bullying-related role adoption, a dynamic approach was adopted which relates changes in teacher interventions across the school year to future bullying-related role adoption. Importantly, teacher interventions were empirically determined based on students’ reports, while bullying roles were assessed via peer nominations. It was hypothesized that different teacher interventions have differential effects on the likelihood of students adopting bullying-related roles. Regarding non-intervention, it was hypothesized that higher levels of non-intervention early in the school year and/or an increase in non-intervention through the school year would increase the chances of adopting the roles of bully, victim, or bully-victim, but decrease the chances of becoming a defender, because students might model the teacher’s behavior of not intervening. Higher scores early in the school year or an increase throughout the school year in disciplinary sanctions were expected to reduce the likelihood of being a bully, a victim, and a bully-victim. Regarding group discussion, higher scores early in the school year or an increase throughout the school year were expected to be associated with a higher probability of being a defender and a lower probability of being a bully at the end of the school year. Although mediation/victim support is positively associated with the well-being of victims, it is unclear whether this strategy is able to reduce the likelihood of being a bully, a victim, a bully-victim, or a defender. No specific hypotheses were formulated for this intervention strategy.

## Methods

### Participants

In two data collection waves, this study surveyed 750 (50.5% female) early adolescent students (7th grade) from a random sample of 20 elementary schools in Prague, Czech Republic. Students were clustered in 39 classes, with the number of classes per school ranging from 1 to 4 and the number of students per class ranging from 11 to 30. To be included in the sample, students had to provide written parental consent and voluntary informed consent. Students were not compensated for their participation. At wave 1, almost all students reported having reached the age of 12 (52.8%) or 13 (44.3%) years (*M*_age_ = 12.9, *SD* = 0.40; range: 11–15; 1.7% did not state their age). Regarding ethnicity, 88.1% described themselves as Czech, 10.4% as non-Czech (1.5% as Vietnamese, 0.5% as Roma, 8.5% as not specified), and 1.5% did not provide their ethnicity.

### Procedure

In order to obtain a representative sample of Prague elementary schools, 28 schools were randomly selected and invited to participate in the study. Overall, 20 out of these schools agreed to cooperate. There were no differences in school size between the schools that accepted the invitation and those that declined it. Data were collected in all seventh-grade classrooms in two waves approximately six months apart during the same school year, with the final wave occurring at the end of the school year. This interval is optimal because it does not confound results, as teacher-induced changes in bullying-related role adoption need time to evolve and summer breaks per se have been found to have similar effects as anti-bullying interventions (e.g., Strohmeier et al., 2010).

Students completed the paper-pencil questionnaires during school hours. To ensure privacy and reduce social desirability, teachers were absent throughout the process; this was considered particularly important because students were asked about their teachers’ behavior. Students were guided and overseen by a trained research team following a standardized protocol. To match student data across the two waves and store their data anonymously, each student was assigned a unique ID number. At the end, students were thanked and provided with a leaflet with phone numbers of anti-bullying helplines and general information on how to cope effectively with bullying. The present research was approved by the Ethics Committee of the third author’s institution, the Czech Academy of Sciences.

### Participation Rates and Missing Data

Student attendance rate relative to all registered 910 seventh-graders was 76.8% (699) in wave 1, and 72.7% (662) in wave 2; 67% (610) of students attended in both waves. Students who provided parental and personal consent, but participated only in one wave could be fully included in the study; this was possible because the present study used only predictors that are either stable across time (gender, ethnicity), rated by peers, or aggregated at the class level. Data from students not providing parental or personal consent were not available (159 students, 17.5%). Across both waves, a total of 751 (82.5%) students were included.

The percentage range of missing values for gender was 0.0%, for ethnicity 1.5%, across the 18 variables measuring teacher interventions 8.0–10.1% for wave 1 and 12.7–13.9% for wave 2, and across the nine variables measuring bullying-related behavior (bullying, victimization, defending) 0.0% for wave 1 and 0.5% for wave 2. The maximum percentage of missing values within school classes across all variables was 22.7%.

When calculating individual peer rating scores (e.g., aggregate of ratings of classmates regarding one person) and aggregate class scores (e.g., class aggregate measuring class-specific teacher interventions), only available ratings were used. To account for missing values for both predictor and outcome variables in the multilevel generalized linear models, full information maximum likelihood (FIML) estimation was used.

### Student-Level Measures

#### Demographic information of students

Students were asked to provide their gender and ethnicity. Gender was effect-coded (−1 = *male*, 1 = *female*) as to produce non-weighted grand mean intercepts in later analyses. Ethnicity was dichotomized and dummy-coded into *Czech* (0) and *non-Czech* (1) ethnicity.

#### Bullying-related student behavior

Three types of behavior were measured using an adaptation of Pozzoli and Gini’s (2010) peer-rating scale: (1) bullying others, (2) being victimized, and (3) defending others. The items were translated into the Czech language and culturally adapted. Students were asked to rate all other classmates on how often they had behaved in a certain way in the past two to three months. Each scale consisted of three items representing physical, verbal, and relational aspects with the answer options *Never* (1), *Sometimes* (2) and *Often* (3). For the bullying scale, the items were “Hits or pushes some classmates”, “Offends or gives nasty nicknames to some classmates”, and “Excludes some classmates from the group or spreads rumors when they do not hear him/her”. For the victimization scale, the items were “Some classmates attack, hit or push him/her”, “Some classmates give him/her nasty nicknames or offend him/her”, and “Some classmates spread nasty rumors about him/her”. For the defending scale, the items were “Defends classmates who are hit or pushed by others”, “Attempts to stop a classmate, who teases or threatens somebody from the classroom”, and “Attempts to help or comfort classmates who are at the margin of the group or excluded from it”.

Students were asked to provide a rating for all of their classmates (Mehari et al., 2019). Peer-rating scores for each student were computed by calculating the mean of all available ratings regarding the respective student by their classmates. Internal consistencies were excellent (all αs > 0.85; see Table 1 for descriptive statistics). Peer-ratings were used to identify bullying-related roles (see Result section).

**Table 1:** Descriptive statistics for the continuous student-level scales at both waves

	Wave 1			Wave 2		
Scale	Range	*M (SD)*	Cronbach α	Range	*M (SD)*	Cronbach α
Bullying-related student behavior						
Bullying (3 items)	1.00–2.57	1.28 (0.28)	0.889	1.00–2.54	1.29 (0.27)	0.878
Victimization (3 items)	1.00–2.27	1.19 (0.19	0.884	1.00–2.14	1.19 (0.19)	0.856
Defending (3 items)	1.00–2.36	1.47 (0.24)	0.907	1.02–2.36	1.42 (0.24)	0.933
Bullying-related teacher interventions						
Non-intervention (2 items)	1.00–5.00	1.93 (0.96)	0.779	1.00–5.00	2.05 (0.98)	0.786
Disciplinary sanction (3 items)	1.00–5.00	4.14 (0.82)	0.736	1.00–5.00	4.13 (0.89)	0.808
Group discussion (4 items)	1.00–5.00	3.67 (1.01)	0.858	1.00–5.00	3.60 (1.09)	0.880
Mediation/victim support (9 items)	1.00–5.00	3.90 (0.78)	0.902	1.00–5.00	3.76 (0.88)	0.924

Note. *N* = 750

### Class-Level Measures

#### Demographic classroom composition

Gender and ethnicity composition of the classroom were measured by the percentage of female and non-Czech students, respectively.

#### Bullying-role related classroom composition

Bullying roles were aggregated for each class, yielding five variables: percentage of bullies, victims, bully-victims, defenders, and non-participants. In order to avoid multicollinearity, percentage of non-participants were not included into regression-based analyses.

#### Teacher bullying-related interventions

Teacher interventions were measured using an adapted 26-item scale by Campaert et al. (2017). Students were asked to indicate on a scale ranging from *Never* (1) to *Always* (5) how often their teachers responded with particular intervention strategies “when a classmate is bullying someone” (bullying scale; 11 items) and “when a classmate is being bullied” (victimization scale; 11 items). Both scales are divided into four subscales, with the first three subscales being the same for both scales: *Non-intervention* (3 items), *Group discussion* (2 items), and *Mediation* (3 items). The bullying scale also included the subscale *Disciplinary sanctions* (3 items), and the victimization scale the subscale *Victim support* (3 items). Two of the three items in both scales’ *Non-intervention* subscales were reverse worded (i.e., “The teacher intervenes.” and “The teacher is aware of the problem.”) and led to substantial cross-loadings in a factor analysis. These two items were excluded in subsequent analyses. Internal consistencies of all scales were good (all αs ≥ 0.74). Descriptive statistics at the student level are presented in Table 1 (for item wording see Supplemental Table S1). In order to obtain a class-level measure of teacher interventions, ratings were averaged across all students of each class.

### Plan of Analysis

Factor analyses with principal component extraction (eigenvalue > 1) and Oblimin (δ = 0) rotation with Kaiser normalization were performed for teacher interventions separately for both waves (see Supplemental Table S1).

In line with previous studies (e.g., Lee et al., 2017) students were assigned to bullying-related roles by standardizing their peer-ratings for bullying, victimization and defending. The overall mean and SD across both wave 1 and wave 2 were used to ensure that students with the same value at both wave 1 and wave 2 were assigned to the same student group. Students with a *z*-score larger than one on the bullying peer ratings (and *z* < 1 on the victimization peer ratings) were identified as (pure) bullies, and those with a *z*-score larger than one on the victimization peer ratings (and *z* < 1 on the bullying peer ratings) were identified as (pure) victims. Those with a *z*-score larger than one on both the bullying and victimization peer ratings were identified as bully-victims. The remaining students were either categorized as defenders (*z* < 1 on both victimization and bullying peer ratings, but *z* > 1 on defender ratings) or being non-participants (all three: *z* < 1). Categorization was exhaustive and exclusive; every student was assigned to exactly one role.

To answer the main research questions, multilevel multinomial logistic regression analyses (Heck & Thomas, 2015) were modelled to account for the nested data structure using Mplus Version 8 (Muthén & Muthén, 2017). Missing information on both dependent and independent variables were handled with full information maximum likelihood (FIML) estimation by including the intercepts of all predictors in the model. All three models used a robust maximum likelihood estimator (referred to as MLR in Mplus). Before estimating the models, zero-level correlations of all model variables were inspected.

Firstly, Model 0 with the cluster variable school class and the (unordered) nominal outcome variable bullying-related role at wave 2 (values: bully, victim, bully-victim, defender, non-participant; the latter being the reference category) was specified to determine the relative proportion of the variance of the dependent variable at student and class level. Secondly, two more elaborate models were calculated (Fig. 1). In order to investigate the static longitudinal effect of teacher interventions at wave 1 on the likelihood to adopt bullying-related roles at wave 2, Model 1 was estimated by including predictors (teacher interventions) at class level and control variables (gender, ethnicity, and student roles at wave 1) at the student and class level (Saarento et al., 2015). Age was not controlled for because of its homogeneity (students were in seventh grade). To investigate the longitudinal dynamic effect of change in teacher interventions on the likelihood of adopting bullying-related roles, Model 2 was estimated. The only difference between Model 1 and Model 2 is that Model 2 used change in teacher interventions (change scores: wave 2 – wave 1) as predictors instead of teacher interventions at wave 1. This model did not adjust for teacher interventions at wave 1, because methodological studies warned that this might lead to erroneous (Sorjonen et al., 2019) and inflated (Edwards, 2002) results.

## Results

### Teacher Interventions Against Bullying

In order to test whether a more efficient and parsimonious measure across bullying and victimization scales is possible, all 26 items of the adapted and translated scale by Campaert et al. (2017) were subjected to an exploratory factor analysis using Oblimin rotation. This was done separately for wave 1 and wave 2. After excluding two items because of cross-loadings, factor analysis for wave 1 yielded 4 factors (explaining 66.7% of total variance), collapsing *mediation* and *victim support* into one scale. A factor analysis at wave 2 yielded the same four-factor solution (explaining 70.9% of the variance). Factor loadings for both waves can be found in Supplemental Table S1. Descriptive statistics at the student level (including reliabilities) are presented in Table 1.

### Prevalence Rates of Bullying-Related Roles Separated by Gender and Wave

For both waves, the majority of students were categorized as *non-participants*, being followed by *defenders, bully-victims, bullies*, and *victims* (see Supplemental Table S2 for bullying-related role frequencies and percentages separated by gender, and Supplemental Table S3 for a cross-tabulation of both waves).

### Differential Effects of Teacher Interventions on Bullying-Related Role Adoption

Before being included in the multilevel multinomial logistic regression analyses, the study variables were correlated at the student and class levels. At the class level, a higher proportion of bullies in the class was associated with more group discussion at wave 1; a higher class proportion of bully-victims with a decline in mediation/victim support from wave 1 to wave 2; and a higher proportion of defenders in the class with more mediation/victim support at wave 1. All these correlations were of moderate size (i.e. between 0.25 and 0.35; for more correlations, see Table 2).

**Table 2:** Correlation coefficient (below the diagonal) and variance (diagonal) matrices for all study variables for student and class level separately

Student-level variables	1	2	3	4	5	6	7	8	9	10
1. Gender (1 = female)	1.000									
2. Ethnicity (1 = non-Czech)	0.112**	0.090								
3. T1: Bully	−0.252***	−0.024	0.063							
4. T1: Victim	−0.046	0.062	−0.062***	0.047						
5. T1: Bully-victim	−0.198***	<0.001	−0.085***	−0.072***	0.082					
6. T1: Defender	0.198***	−0.014	−0.110***	−0.093***	−0.129***	0.122				
7. T2: Bully	−0.219***	−0.005	0.436***	−0.015	0.166**	−0.099***	0.067			
8. T2: Victim	−0.029	0.028	−0.021	0.464***	−0.016	−0.067**	−0.068***	0.053		
9. T2: Bully-victim	−0.165***	−0.023	0.052	0.046	0.603***	−0.110***	−0.084***	−0.073***	0.076	
10. T2: Defender	0.196***	0.016	−0.090***	−0.013	−0.089***	0.613***	−0.093***	−0.082***	−0.100***	0.090

Class-level variables	1	2	3	4	5	6	7	8	9	10	11	12	13	14
1. Gender (% female in class)	2.101													
2. Ethnicity (% non-Czech in class)	−0.035	0.464												
3. T1: Bullies (% in class)	−0.327**	0.167	0.472											
4. T1: Victims (% in class)	0.125	0.281	0.075	0.294										
5. T1: Bully-victims (% in class)	−0.244	0.026	−0.061	0.271	1.549									
6. T1: Defenders (% in class)	0.370*	−0.079	−0.182	0.089	0.020	3.950								
7. T1: Teachers: No intervention	0.166	0.073	−0.185	0.060	−0.145	−0.013	0.101							
8. T1: Teachers: Disciplinary sanction	0.036	−0.144	0.197	−0.064	−0.131	0.206	−0.602***	0.085						
9. T1: Teachers: Group discussion	−0.223*	0.057	0.275*	0.129	0.016	−0.001	−0.413**	0.495***	0.121					
10. T1: Teachers: Mediation/victim support	−0.080	−0.030	0.200	0.184	0.172	0.325*	−0.678***	0.691***	0.605***	0.086				
11. Δ T2 − T1: No intervention	−0.173	−0.257	0.180	−0.161	0.117	−0.098	−0.256	0.070	−0.129	0.129	0.062			
12. Δ T2 − T1: Disciplinary sanction	0.237	0.265	−0.186	0.091	0.011	−0.002	0.380*	−0.592***	−0.017	−0.461**	−0.339*	0.083		
13. Δ T2 − T1: Group discussion	0.185	0.339*	−0.064	0.061	−0.149	−0.056	0.150	−0.199	−0.192	−0.256*	−0.080	0.238	0.136	
14. Δ T2 − T1: Mediation/victim support	0.182	0.096	−0.197	−0.221	−0.271*	−0.157	0.164	−0.274	0.052	−0.401**	−0.315*	0.517***	0.449***	0.047

*N* = 750; Values below the diagonal are correlation coefficients; values on the diagonal are variances

On the student-level, gender is effect-coded (–1 = *male*, 1 = *female*), all other variables are dummy coded; the reference category for bully, victim, bully-victim and defender is non-participant. On the class-level, variables 1–6 refer to class composition, and represent class percentages, whereas 1 unit represent 10% (possible range: 0–10); variables 7–10 refer to class-aggregated teacher intervention scores and have been grand-mean centered; variables 11–14 represent grand-mean-centered difference scores between class-aggregated teacher intervention scores (wave 2 − wave 1)

*p ≤ 0.05, **p ≤ 0.01, ***p ≤ 0.001

The empty model (Model 0; see Supplemental Table S4) showed that all intraclass correlation coefficients differed significantly from zero, indicating heterogeneity of bullying-related roles between school classes, with between-classes variance accounting for 16–64% of overall variance in the likelihood of adopting a specific role (see Supplemental Table S4). These results indicate that multilevel modelling is needed to avoid bias due to failure to account for classroom clusters.

Both the static model (Model 1) and the dynamic model (Model 2) showed multiple significant effects of teacher interventions and teacher intervention change on bullying-related role adoption at wave 2 while controlling for student- and class-level background variables. The estimates are log odds, interpreted relative to the reference category (non-participants).

Regarding the static model (see Table 3), higher levels of disciplinary sanctions at wave 1 were associated with lower odds of being a bully or a victim at wave 2. Higher levels of teacher mediation/victim support were associated with higher odds of being a bully. Higher levels of group discussion were associated with higher odds of being a defender.

**Table 3:** Model 1 (static): Results of a multilevel multinomial logistic regression analysis predicting student roles at wave 2 by teacher interventions while controlling for several demographic and bullying-related factors on the student- and class level

Variable	T2: Bully vs. non-participant				T2 Victim vs. non-participant				T2 Bully-victim vs. non-participant				T2 Defender vs. non-participant			
	*Est*	*SE*	*p*	*OR*	*Est*	*SE*	*p*	*OR*	*Est*	*SE*	*p*	*OR*	*Est*	*SE*	*p*	*OR*
Student level																
Gender (1 = female)	−0.578*	0.227	0.011	0.56	0.060	0.229	0.793	1.06	−0.289	0.216	0.181	0.75	0.524*	0.225	0.020	1.69
Ethnicity (1 = non-Czech)	0.329	0.629	0.601	1.39	−0.112	0.606	0.853	0.89	−0.264	0.683	0.699	0.77	0.546	0.586	0.352	1.73
Role (reference: non-participant)																
- Bully	3.578***	0.436	<0.001	35.79	0.631	0.710	0.375	1.88	2.375***	0.573	<0.001	10.76	−6.906***	0.461	<0.001	0.00
- Victim	2.306**	0.815	0.005	10.03	4.681***	0.637	<0.001	107.91	3.443***	0.984	<0.001	31.28	3.662***	0.931	<0.001	38.95
- Bully-victim	4.048***	0.629	<0.001	57.29	2.604***	0.703	<0.001	13.52	4.942***	0.817	<0.001	140.06	2.045	1.114	0.066	7.73
- Defender	−0.287	1.281	0.822	0.75	−0.586	0.903	0.517	0.56	−1.088	1.039	0.295	0.34	3.069***	0.435	<0.001	21.52
CLASS LEVEL																
Gender (% female)	−0.372*	0.184	0.043	0.69	−0.035	0.198	0.859	0.97	0.039	0.249	0.875	1.04	0.176	0.190	0.352	1.19
Ethnicity (% non-Czech)	−0.362	0.369	0.326	0.70	0.041	0.307	0.894	1.04	−0.429	0.436	0.326	0.65	−0.011	0.401	0.977	0.99
Class role percentage (T1)																
- Bullies (%)	0.356	0.311	0.252	1.43	1.248***	0.350	<0.001	3.48	1.124**	0.403	0.005	3.08	0.988**	0.379	0.009	2.69
- Victims (%)	−0.084	0.413	0.839	0.92	0.213	0.539	0.693	1.24	0.659	0.584	0.259	1.93	−1.739**	0.615	0.005	0.18
- Bully-victims (%)	−0.082	0.173	0.636	0.92	0.461**	0.150	0.002	1.59	0.466*	0.228	0.040	1.59	0.613***	0.171	<0.001	1.85
- Defenders (%)	−0.156	0.179	0.383	0.86	−0.228	0.254	0.368	0.80	0.464*	0.185	0.012	1.59	0.134	0.273	0.623	1.14
Teacher interventions (T1)																
- Non-intervention	1.799	1.045	0.085	6.04	−1.172	0.621	0.059	0.31	−2.131	1.319	0.106	0.12	0.133	1.153	0.908	1.14
- Disciplinary sanction	−2.904**	1.095	0.008	0.05	−2.874*	1.313	0.029	0.06	−2.132	1.621	0.188	0.12	−2.400	1.740	0.168	0.09
- Group discussion	−0.520	0.720	0.470	0.59	−0.758	0.883	0.391	0.47	0.190	1.098	0.863	1.21	2.523*	1.171	0.031	12.47
- Mediation/victim support	4.094**	1.321	0.002	59.98	1.018	1.497	0.497	2.77	−1.520	2.038	0.456	0.22	−0.762	1.769	0.667	0.47
Intercept	−3.974***	0.328	<0.001	0.02	−3.561***	0.394	<0.001	0.03	−4.201***	0.480	<0.001	0.01	−4.119***	0.509	<0.001	0.02
Residual variance	0.133	0.257	0.604	–	0.333	0.436	0.445	–	0.705*	0.345	0.041	–	1.926*	0.864	0.026	–
Intraclass correlation (ICC)	0.039	0.072	0.589	–	0.092	0.109	0.401	–	0.176*	0.071	0.013	–	0.369***	0.104	<0.001	–

Student level *N* = 750; class level *N* = 39; *AIC* = 2812.752; *BIC* = 3265.519, Loglikelihood H_0_ = − 1308.376, H_0_ Scaling correction factor for robust maximum likelihood estimation (MLR) = = 1.7254. At the student level where the outcome variable is multinomial (student roles), raw estimates (*Est*) represent log odds. At the class level where the outcome variable is continuous (latent random intercept), estimates represent linear regression slopes. These can be interpreted as log-odds when the log of the odds of student role adoption at student level is considered to be the dependent variable). On the student-level, gender is effect-coded (−1 = *male*, 1 = *female*), all other variables are dummy coded; ethnicity has been group-centered; the reference category for student roles is non-participant. On the class-level, the first six variables refer to class composition, and represent class percentages, whereas 1 unit represent 10% (possible range: 0–10); the remaining variables refer to class-aggregated teacher intervention scores and have been grand-mean centered

*p ≤ 0.05, **p ≤ 0.01, ***p ≤ 0.001

Regarding the dynamic model (see Table 4), a decrease of general intervention across time (i.e., increasing non-intervention) was associated with higher odds of being a victim and lower odds of being a defender at wave 2. An increase in group discussion across time was associated with higher odds of being a defender.

**Table 4:** Model 2 (dynamic): Results of a multilevel multinomial logistic regression analysis predicting student roles at wave 2 by change in teacher interventions while controlling for several demographic and bullying-related factors on the student- and class level

Variable	T2: Bully vs. non-participant				T2 Victim vs. non-participant				T2 Bully-victim vs. non-participant				T2 Defender vs. non-participant			
	*Est*	*SE*	*p*	*OR*	*Est*	*SE*	*p*	*OR*	*Est*	*SE*	*p*	*OR*	*Est*	*SE*	*p*	*OR*
Student level																
Gender (1 = female)	−0.557**	0.195	0.004	0.57	0.098	0.232	0.674	1.10	−0.285	0.217	0.188	0.75	0.585*	0.250	0.019	1.80
Ethnicity (1 = non-Czech)	0.314	0.580	0.589	1.37	−0.400	0.770	0.603	0.67	−0.334	0.725	0.645	0.72	0.460	0.606	0.448	1.58
Role (reference: non-participant)																
- Bully	3.308***	0.451	<0.001	27.34	0.437	0.797	0.583	1.55	2.338***	0.533	<0.001	10.36	−6.505***	0.542	<0.001	0.001
- Victim	1.972*	0.800	0.014	7.18	4.707***	0.601	<0.001	110.67	3.285***	0.934	<0.001	26.71	3.535***	0.837	<0.001	34.30
- Bully-victim	3.788***	0.588	<0.001	44.17	2.449***	0.700	<0.001	11.57	4.891***	0.789	<0.001	133.10	2.151	1.150	0.061	8.60
- Defender	−0.363	1.257	0.773	0.70	−0.780	0.997	0.434	0.46	−0.905	1.007	0.369	0.41	3.317***	0.374	<0.001	27.59
CLASS LEVEL																
Gender (% female)	−0.282	0.187	0.132	0.75	−0.075	0.245	0.759	0.93	0.085	0.199	0.668	1.09	−0.060	0.200	0.763	0.94
Ethnicity (% non-Czech)	−0.304	0.444	0.494	0.74	0.308	0.353	0.383	1.36	−0.431	0.468	0.358	0.65	−0.456	0.399	0.253	0.63
Class role percentage (T1)																
- Bullies (%)	0.532	0.355	0.134	1.70	0.838*	0.353	0.018	2.31	1.105*	0.459	0.016	3.02	1.383**	0.463	0.003	3.99
- Victims (%)	0.634	0.543	0.243	1.89	−0.055	0.497	0.912	0.95	0.322	0.460	0.484	1.38	−1.169*	0.559	0.036	0.31
- Bully-victims (%)	0.186	0.149	0.213	1.20	0.410**	0.145	0.005	1.51	0.513**	0.198	0.010	1.67	0.630***	0.131	<0.001	1.88
- Defenders (%)	−0.047	0.178	0.790	0.95	−0.022	0.255	0.932	0.98	0.668***	0.167	<0.001	1.95	0.289	0.303	0.340	1.34
Δ Teacher interventions (T2 − T1)																
- Δ Non-intervention	−1.163	1.279	0.363	0.31	2.388*	1.144	0.037	10.89	−1.245	1.252	0.320	0.29	−2.269*	1.027	0.027	0.10
- Δ Disciplinary sanction	−0.062	1.091	0.954	0.94	1.545	1.096	0.159	4.69	−1.424	0.919	0.121	0.24	−1.253	1.834	0.495	0.29
- Δ Group discussion	−0.171	0.610	0.779	0.84	0.345	0.734	0.638	1.41	1.560	0.813	0.055	4.76	1.546*	0.688	0.025	4.69
- Δ Mediation/victim support	0.449	1.342	0.738	1.57	0.116	1.144	0.919	1.12	0.642	1.586	0.686	1.90	2.302	2.190	0.293	9.99
Intercept	−4.015***	0.433	<0.001	0.02	−3.619***	0.35	<0.001	0.03	−4.462***	0.54	<0.001	0.01	−4.622***	0.515	<0.001	0.01
Residual variance	0.503	0.396	0.204	–	0.266	0.341	0.435	–	0.577	0.441	0.191	–	1.016*	0.483	0.036	–
Intraclass correlation (ICC)	0.133	0.091	0.143	–	0.075	0.089	0.399	–	0.149	0.097	0.124	–	0.236**	0.086	0.006	–

Student level *N* = 750; class level *N* = 39; *AIC* = 2777.382; *BIC* = 3230.149, Loglikelihood H_0_ = − 1290.691, H_0_ Scaling correction factor for robust maximum likelihood estimation (MLR) = 1.6880. At the student level where the outcome variable is multinomial (student roles), raw estimates (*Est*) represent log odds. At the class level where the outcome variable is continuous (latent random intercept), estimates represent linear regression slopes. These can be interpreted as log-odds when the log of the odds of student role adoption at student level is considered to be the dependent variable). On the student-level, gender is effect-coded (−1 = *male*, 1 = *female*), all other variables are dummy coded; ethnicity has been group-centered; the reference category for student roles is non-participant. On the class-level, the first six variables refer to class composition, and represent class percentages, whereas 1 unit represent 10% (possible range: 0–10); the remaining variables refer to class-aggregated teacher intervention change scores and have been grand-mean centered

*p ≤ 0.05, **p ≤ 0.01, ***p ≤ 0.001

## Discussion

Bullying in schools is a widespread phenomenon that hinders the healthy development of students. Teacher interventions play an important role in keeping schools healthy and safe, but to date it is unclear which teacher interventions are actually effective in achieving lasting changes in bullying-related student behavior. This study extends the literature by establishing that different teacher interventions have distinct effects on the adoption of specific bullying-related roles in early adolescent students within one school year, over a period of six month. The most important finding of this study is that disciplinary sanctions and group discussions showed beneficial effects on students’ role adoption over time, while non-intervention and mediation/victim support had a higher risk of being followed by non-beneficial changes in role adoption. The study underscored that it is necessary to devote more attention to the highly vulnerable group of bully-victims (Yang et al., 2016), as no effective teacher interventions were found for this group.

### Beneficial Effects of Disciplinary Sanctions and Group Discussion

Imposing disciplinary sanctions by clearly communicating to the bully that the behavior was unacceptable and reporting the event to other adults (e.g., school principal or parents) turned out to be the most effective intervention strategy in directly tackling bullying and victimization. In the static longitudinal model, higher levels of disciplinary sanctions were shown to decrease the likelihood of being a victim or being a bully over time. This result is in line with previous studies showing that disciplining the bully might be an effective measure to reduce bullying (Gaffney et al., 2021). The found associations support the notion that disciplinary sanctions should be considered an essential component of anti-bullying interventions (Garandeau et al., 2021; Limber et al., 2018; Strohmeier et al., 2021). It must be pointed out that in the present study disciplinary measures were operationalized of low to moderate severity and more severe measures (e.g., detention, suspension, and expulsion) were not included.

The second most effective teacher intervention was teacher-facilitated group discussion. This was the only intervention that showed beneficial effects in both the static and the dynamic longitudinal model: Higher levels of group discussion early in the school year or an increase in discussions through the school year raised the likelihood of being a defender. Although group discussion did not directly reduce the likelihood of being a bully or a victim, it mobilized bystanders (the silent majority) to be more likely to defend, which might indirectly lead to a cascade of self-reinforcing positive developments (Saarento et al., 2013). Regardless of whether defending stops bullying or not, it may reduce some of the negative consequences of victimization by helping victimized students maintain a certain level of self-worth and social acceptance by peers (Sainio et al., 2010).

### Adverse Effects of Non-Intervention and Mediation/Victim Support

An increase in non-intervention showed unfavorable effects by increasing the likelihood of being a victim and decreasing the likelihood of being a defender. The fact that non-intervention promotes the victim role and discourages the defender role is consistent with previous research that shows that teachers influence student behavior through social modeling (Saarento et al., 2015). Non-intervention by teachers might reinforce bullies by conveying the message that victimizing other students has no negative consequences (Mucherah et al., 2018) and bullies might perceive their behavior as acceptable. When teachers do not respond to bullying, students seem to be less likely to defend victims. These findings are alarming and must be taken seriously. Teacher education should raise awareness among teachers that not intervening is harmful.

It is important to note that not all teacher interventions are appropriate for bringing about positive changes in bullying-related group adoption. Mediation/victim support had no beneficial effects on bullying-related role adoption. Paradoxically, it even had an iatrogenic effect of promoting being a bully. This could be due to the fact that teachers who use mediation and/or victim support may fail to communicate clear boundaries of acceptable behavior to the bullies, who subsequently are not required to take responsibility and may continue with their problematic behavior unhindered and with impunity. This is consistent with the rationale of many effective anti-bullying programs (e.g., KiVa, OBPP and ViSC), which is that targeting the behaviors of bullies is key to reducing bullying, while talking to victims is key to increasing their well-being and reassuring them that the teacher is there to help them. Although being largely used in tandem in the present sample, mediation and victim support might have different outcomes when used independently from each other. While mediation may be an appropriate strategy for resolving normal conflicts, it is not appropriate for bullying cases due to the pronounced power imbalance between bullies and victims (Rigby, 2012). The teachers’ impartial position could create the impression that both bullies and victims contributed equally to the problem and that bullies do not need to fear consequences for their behavior (Limber, 2017). Victim support, on the other hand, might help the victims feel less isolated, better endure the bullying, and might even mitigate some negative effects on the victims’ health (Yeung & Leadbeater, 2010). In the present study, the combination of the two strategies was not sufficient to stop the bullying and to help the victim escape the bullying situation (Rigby, 2012).

### No Effects on Bully-Victims

In both the static and the dynamic model, neither teacher interventions nor teacher intervention change had an effect on the likelihood of being a bully-victim. This is concerning because bully-victims, as a high-risk group, have been shown to be particularly likely to experience negative effects on their psychological well-being and health (Yang et al., 2016). Findings from previous studies indicate that this group of students is difficult to address through interventions (Sung et al., 2020). One reason for this might be that bully-victims use bullying other more vulnerable students as a maladaptive coping strategy that might seem particularly functional to them and is maintained by them (Fischer et al., 2022). Other reasons might be that this group of students includes individuals with different developmental processes (Ettekal & Ladd, 2020) and bullying-related role trajectories (e.g., Sung et al., 2018) and is rather heterogeneous (Kennedy, 2021).

It has been shown that bully-victims need the help of teachers because of their serious situation, but teachers have great difficulty in supporting them (Berkowitz & Benbenishty, 2012) because teachers might perceive them as only perpetrators. Bully-victims need a complex approach that includes both setting clear boundaries regarding their bully role and providing teacher support regarding their victim role (Sung et al., 2020). The present findings call for future intervention research and point to the need to foster teacher readiness to work with bully-victims and, when needed, to implement interventions going beyond teacher interventions (e.g., involving peers, parents, psychologists). Multimodal intervention programs targeting this vulnerable group could focus on managing anger, eliminating hostile attribution bias, and promoting prosocial behavior strategies (e.g., Strohmeier et al., 2021).

### Methodological Considerations

The methodology used is a strength of this study. Importantly, the static and the dynamic analytical approach provided complementary information (Nguyen et al., 2020). The static model revealed beneficial effects of disciplinary sanctions and group discussion as well as the unfavorable effects of mediation/victim support. The dynamic model showed the beneficial effects of an increase of group discussion and the unfavorable effects of an increase in non-intervention throughout the school year. The only teacher strategy that had an effect in both models was group discussion. Future research should use both static and dynamic models, as they complement each other. It is important to know not only whether teachers take action early in the school year, but also whether they maintain their actions over time, and what impact is associated with each of these patterns.

The present study demonstrated that a person-centered approach (Burger & Bachmann, 2021) is ideally suited to investigate differential effects of teacher interventions on the adoption of functionally distinct bullying-related roles. The study took advantage of differences in teacher and student behavior that occurred during one school year, over a six-month period under real-world conditions. This real-world approach avoids artificial results that do not align with the realities of everyday school life, ensuring robust external validity (Leatherdale, 2018).

### Practical Implications

This study yielded promising results that help identify intervention strategies that work for youth in early adolescence, a developmental period when bullying-related behavior peaks and students are particularly vulnerable due to biological, mental, and social changes (Troop-Gordon, 2017). The findings demonstrate that specific intervention strategies are especially effective for specific bullying-related roles, and in turn, help improve both pre-service teacher training, professional development programs, and antibullying programs. Based on the findings, intervention programs should include both group discussion and (moderate) disciplinary action, as these two strategies turned out to be the intervention strategies with the most beneficial effects on role adoption over time. In the present study, mediation/victim support was not followed by a lower likelihood of being a victim. It is important to emphasize that these findings should not be taken to suggest that teachers should refrain from supporting victims. Although this strategy might not help victims escape their role, previous research has shown that victim support mitigates some of the negative effects of bullying on victims. It should not be viewed as a stand-alone strategy to stop bullying but as a complementary strategy aimed at improving the adjustment of victimized students (Rigby, 2012).

### Limitations and Recommendations for Future Research

Despite important strengths, such as the longitudinal multi-level design, the multi-informant measurement of relevant study variables, the person-oriented methods, and the combination of the static and the dynamic analytical approach, this study has some limitations. Firstly, the Czech Republic, where this study was conducted, is one of the EU countries that do not yet have a fully developed network of nationwide evidence-based anti-bullying programs (Miovsky, 2015) and lacks systematic anti-bullying teacher education (Janošová et al., 2016). It is possible that the present results differ from findings examined in countries where national programs and teacher education on bullying have been developing for decades (e.g., Finland; Garandeau et al., 2021). Secondly, effectiveness of teacher interventions might depend on student age (e.g. bullying behavior might become less directly observable with older students; Yeager et al., 2015). Future research should replicate and experimentally validate these findings in other contexts and with different age groups. Thirdly, the measures used may capture mainly the publicly visible parts of teacher and student behavior (Cornell & Bandyopadhyay, 2010), as less conspicuous behaviors may not be noticed by classmates. But it is plausible that classmates know that bullying occurs and teachers intervene even if they are not present, because bullying happens only rarely in secret (Smith et al., 2022) and is likely to be passed on immediately to other students. Future research could focus on differentiating and measuring different forms and types of bullying (e.g. direct forms such as physical bullying, and indirect forms such as relational bullying). Fourthly, in the present sample, victim support and mediation were largely used in tandem and were combined into one factor, which may limit the generalizability of the results. Finally, although the sample was large on the student level, the sample size of 39 classrooms—combined with the fact that bullying-related role groups are relatively small—limited statistical power and made it impossible to account for cross-level interaction effects. With larger sample sizes on the class level, future research should examine more complex models including moderating, mediating or bidirectional processes and should take into account within and between time effects.

## Conclusion

Early adolescence is a developmental period when students are particularly vulnerable to school bullying, which in turn is often accompanied by serious long-term consequences on students’ psychological, social, academic and overall functioning. Teachers play an important role in stopping bullying and preventing these harmful consequences. The extant literature is very limited on the impact of teacher interventions on students’ adoption of bullying-related roles in early adolescence. The present study addressed this research gap by jointly exploring the effects of four teacher intervention strategies (disciplinary sanctions, group discussions, mediation/victim support, non-intervention) on the adoption of four bullying-related roles (bully, victim, bully-victim, defender). Analyses identified two teacher interventions that had beneficial effects on bullying-related role adoption. Disciplinary sanctions reduced the likelihood of being a bully or a victim, and teacher-facilitated group discussions increased the likelihood of being a defender. Adverse effects were found for non-intervention, which increased the likelihood of being a victim and decreased the likelihood of being a defender, and for mediation/victim support, which increased the likelihood of being a bully. The findings evidence the fact that teachers can make a difference in the fight against bullying in early adolescence. The results carry important practical implications for the professional training of prospective and current teachers working with this age group. Teachers are in a unique position and have several routes to shape the social dynamics in a class, and can influence students’ involvement in prosocial or problematic bullying-related roles.

## Supplementary information

Supplementary Information
