# Supplementary material for: Teachers Can Make a Difference in Bullying: Effects of Teacher Interventions on Students’ Adoption of Bully, Victim, Bully-Victim or Defender Roles across Time
Source: J Youth Adolesc. 2022 Sep 2;51(12):2312–27. doi: 10.1007/s10964-022-01674-6 (PMC9596519; doi:10.1007/s10964-022-01674-6)
Supplement: Supplementary file 1 — Supplementary Information [file 10964_2022_1674_MOESM1_ESM.docx]

**Supplemental Table S1**

*Summary of exploratory factor analysis results for the teacher intervention items*

| **Item** | | **Rotated Loadings (Factor Pattern coefficients)** | | | |
| --- | --- | --- | --- | --- | --- |
| **Nr** | **Item wording** | **Non- intervention** | **Disciplinary sanctions** | **Group discussion** | **Mediation/ victim support** |
| v3 | Teacher leaves things up to the students. | 0.887 / 0.833 |  |  |  |
| b3 | Teacher leaves things up to the students. | 0.865 / 0.917 |  |  |  |
| b8 | Teacher imposes disciplinary actions against those who bullied. |  | 0.772 / 0.902 |  |  |
| b9 | Teacher reports the event to another adult, such as the school principal or parents. |  | 0.651 / 0.822 |  |  |
| b7 | Teacher says to those who bullied that the behavior was unacceptable. |  | 0.613 / 0.644 |  |  |
| b5 | Teacher discusses the incident with the whole class. |  |  | 0.912 / 0.933 |  |
| v5 | Teacher discusses the incident with the whole class. |  |  | 0.894 / 0.887 |  |
| b6 | Teacher discusses with the whole class that the behavior was wrong. |  |  | 0.737 / 0.756 |  |
| v6 | Teacher discusses with the whole class on how much suffering this behavior caused to the victim. |  |  | 0.718 / 0.727 |  |
| v10 | Teacher tries to help the victim. (vs) |  |  |  | 0.753 / 0.689 |
| v11 | Teacher comforts the victim. (vs) |  |  |  | 0.750 / 0.718 |
| b11 | Teacher helps the involved students to find a solution for the problem. (m) |  |  |  | 0.739 / 0.851 |
| v8 | Teacher helps the involved students to find a solution for the problem. (m) |  |  |  | 0.737 / 0.778 |
| v9 | Teacher tries to have the victim consoled by other students. (vs) |  |  |  | 0.712 / 0.639 |
| v4 | Teacher helps the students resolve the incident. (m) |  |  |  | 0.595 / 0.619 |
| b10 | Teacher tries to make the students involved make peace. (m) |  |  |  | 0.595 / 0.902 |
| v7 | Teacher tries to make the students involved make peace. (m) |  |  |  | 0.578 / 0.777 |
| b4 | Teacher helps the students resolve the incident. (m) |  |  |  | 0.500 / 0.566 |
|  | Total variance (rotation sums of squared loadings) | 3.02 / 3.03 | 4.04 / 5.03 | 4.96 / 5.44 | 6.67 / 7.58 |

*Note.* Principal component analysis was used as extraction method (criterion: eigenvalue > 1) and Oblimin (delta = 0) with Kaiser Normalization as rotation method. The Kaiser-Meyer-Olkin (KMO) measure of sampling adequacy was superb (> 0.90), and Bartlett's test of sphericity was highly significant for the teacher intervention items both at wave 1 (⁠KMO = 0.92; Bartlett χ²(153) = 6163.9; *p* < 0.001) and at wave 2 (⁠KMO = 0.92; Bartlett χ²(153) = 7276.5; *p* < 0.001).

Factor pattern coefficients are analogous to standardized regression coefficients with the effects of the other variables partialled out. Factor-loadings below 0.35 are not represented for the sake of clarity. Values left of the dash pertain to wave 1 (*N* = 615) and right of the dash pertain to wave 2 (*N* = 606).
Items 1 and 2 "Teacher intervenes" and "Teacher is aware of the problem" for both victimization and bullying have been dropped due to cross-loadings with the factors victim support and disciplinary action—the loadings presented here represent a solution without including those items. Items with "b" in the item number were introduced by the following sentence "when a classmate bullies, what does your teacher do?" and those with "v" by "when a classmate is being victimized, what does your teacher do".
(vs) = was part of the subscale *victim support*; (m) = was previously part of the subscale *mediation.*

**Supplemental Table S2**

*Crosstabulation for bullying role and gender at both wave 1 and wave 2 separately*

|  |  | **Wave 1  (*N* = 750)** | | |  | **Wave 2  (*N* = 746)** | | |
| --- | --- | --- | --- | --- | --- | --- | --- | --- |
| **Student Roles** |  | **Female** | **Male** | **Total** |  | **Female** | **Male** | **Total** |
| Bully |  | 2 (0.5%) | 49 (13.2%) | 51 (6.8%) |  | 6 (1.6%) | 48 (13.0%) | 54 (7.2%) |
| Victim |  | 15 (4.0%) | 22 (5.9%) | 37 (4.9%) |  | 19 (5.0%) | 23 (6.2%) | 42 (5.6%) |
| Bully-victim |  | 13 (3.4%) | 55 (14.8%) | 68 (9.1%) |  | 14 (3.7%) | 48 (13.0%) | 62 (8.3%) |
| Defender |  | 80 (21.1%) | 27 (7.3%) | 107 (14.3%) |  | 60 (15.9%) | 15 (4.1%) | 75 (10.1%) |
| Non-participant |  | 269 (71.0%) | 218 (58.8%) | 487 (64.9%) |  | 278 (73.7%) | 235 (63.7%) | 513 (68.8%) |
| Total |  | 379 (100%) | 371 (100%) | 750 (100%) |  | 377 (100%) | 369 (100%) | 746 (100%) |

**Supplemental Table S3**

*Crosstabulation for bullying-related student roles and data collection waves*

| **Wave 1:** |  | **Wave 2: Bullying-related roles** | | | | | |
| --- | --- | --- | --- | --- | --- | --- | --- |
| **Bullying-related roles** |  | **Bully** | **Victim** | **Bully-victim** | **Defender** | **Non-participant** | **Total** |
| Bully |  | *25 (49.0%)* | 2 (3.9%) | 7 (13.7%) | 0 (0.0%) | **17 (33.3%)** | 51 (100%) |
| Victim |  | 2 (5.6%) | *19 (52.8%)* | 5 (13.9%) | 3 (8.3%) | **7 (19.4%)** | 36 (100%) |
| Bully-victim |  | 14 (20.9%) | 3 (4.5%) | *41 (61.2%)* | 1 (1.5%) | **8 (11.9%)** | 67 (100%) |
| Defender |  | 1 (0.9%) | 2 (1.9%) | 1 (0.9%) | *59 (55.1%)* | **44 (41.1%)** | 107 (100%) |
| Non-participant |  | **12 (2.5%)** | **16 (3.3%)** | **8 (1.6%)** | **12 (2.5%)** | *437 (90.1%)* | 485 (100%) |
| Total |  | 54 (7.2%) | 42 (5.6%) | 62 (8.3%) | 75 (10.1%) | 513 (68.8%) | 746 (100%) |

*Note.* Percentages are calculated relative to group totals at wave 1. Cells on the diagonal (presented in italics) represent portions of students maintaining their roles across time. Cells in the penultimate row (presented in bold) represent portions of students who were non-participants but changed their role at wave 2. Cells in the penultimate column (also presented in bold) represent portions of students who became non-participants at wave 2, but were members of other student role groups at wave 1.

**Supplemental Table S4**

*Model 0: Null model with school class as cluster variable and bullying-related student role at wave 2 as outcome variable*

| **T1: Variable** |  | **T2: Bully vs. non-participant** | | |  | |  | | **T2 Victim vs. non-participant** | | |  |  | **T2 Bully-victim vs. non-participant** | | | |  | **T2 Defender vs. non-participant** | | | |
| --- | --- | --- | --- | --- | --- | --- | --- | --- | --- | --- | --- | --- | --- | --- | --- | --- | --- | --- | --- | --- | --- | --- |
| CLASS LEVEL |  | *Est* | *SE* | *p* | | *OR* | |  | *Est* | *SE* | *p* | *OR* |  | *Est* | *SE* | *p* | *OR* |  | *Est* | *SE* | *p* | *OR* |
| Intercept |  | **−2.482***** | 0.243 | <0.001 | | 0.08 | |  | **−2.886***** | 0.347 | <0.001 | 0.06 |  | **−2.878***** | 0.395 | <0.001 | 0.06 |  | **−3.094***** | 0.517 | <0.001 | 0.05 |
| Variance |  | 0.610 | 0.351 | 0.082 | | − | |  | **1.439*** | 0.683 | 0.035 | − |  | **3.348**** | 1.290 | 0.009 | − |  | **5.882*** | 2.819 | 0.037 | − |
| Intraclass correlation (ICC) |  | **0.156*** | 0.076 | 0.039 | | − | |  | **0.304***** | 0.100 | 0.002 | − |  | **0.504***** | 0.096 | <0.001 | − |  | **0.641***** | 0.110 | <0.001 | − |

*Note.* Class level *N* = 39; Student level *N* = 746; *AIC* = 1385.168; *BIC* = 1422.086, Loglikelihood H_0_ = −684.584, H_0_ Scaling correction factor for robust maximum likelihood estimation (MLR) = 0.9933.

* *p* ≤ 0.05, ** *p* ≤ 0.01, *** *p* ≤ 0.001; significant estimates are presented in bold.
